# Survey on the Performance of Source Localization Algorithms

**DOI:** 10.3390/s17112666

**Published:** 2017-11-18

**Authors:** José Manuel Fresno, Guillermo Robles, Juan Manuel Martínez-Tarifa, Brian G. Stewart

**Affiliations:** 1 Department of Electrical Engineering, Universidad Carlos III de Madrid, Avda, Universidad, 30, Leganés, 28911 Madrid, Spain; jfresno@ing.uc3m.es (J.M.F.); jmmtarif@ing.uc3m.es (J.M.M.-T.); 2 Department of Electronic and Electrical Engineering, University of Strathclyde, 204 George Street, Glasgow G1 1XW, UK

**Keywords:** source localization, emitter localization, standard least squares, hyperbolic least squares, hyperbolic positioning, maximum likelihood estimator, Bancroft, particle swarm optimization, combined algorithm

## Abstract

The localization of emitters using an array of sensors or antennas is a prevalent issue approached in several applications. There exist different techniques for source localization, which can be classified into multilateration, received signal strength (RSS) and proximity methods. The performance of multilateration techniques relies on measured time variables: the time of flight (ToF) of the emission from the emitter to the sensor, the time differences of arrival (TDoA) of the emission between sensors and the pseudo-time of flight (pToF) of the emission to the sensors. The multilateration algorithms presented and compared in this paper can be classified as iterative and non-iterative methods. Both standard least squares (SLS) and hyperbolic least squares (HLS) are iterative and based on the Newton–Raphson technique to solve the non-linear equation system. The metaheuristic technique particle swarm optimization (PSO) used for source localisation is also studied. This optimization technique estimates the source position as the optimum of an objective function based on HLS and is also iterative in nature. Three non-iterative algorithms, namely the hyperbolic positioning algorithms (HPA), the maximum likelihood estimator (MLE) and Bancroft algorithm, are also presented. A non-iterative combined algorithm, MLE-HLS, based on MLE and HLS, is further proposed in this paper. The performance of all algorithms is analysed and compared in terms of accuracy in the localization of the position of the emitter and in terms of computational time. The analysis is also undertaken with three different sensor layouts since the positions of the sensors affect the localization; several source positions are also evaluated to make the comparison more robust. The analysis is carried out using theoretical time differences, as well as including errors due to the effect of digital sampling of the time variables. It is shown that the most balanced algorithm, yielding better results than the other algorithms in terms of accuracy and short computational time, is the combined MLE-HLS algorithm.

## 1. Introduction

There is great interest in localizing radiative sources fast and accurately with receivers in many fields of work. Global positioning systems (GPS) are widely used in many applications like navigation, control of autonomous road vehicles [1] and unmanned aerial vehicles [2]. In sonar, radar and underwater radar, it is often of interest to determine the location of an object from its emissions [3,4]. Indoor localization in wireless networks is addressed relying on a swarm-based approach [5]. Earthquake epicentre localization can also be handled with this approach [6,7]. Locating defective assets in electrical facilities through the measurement of the electromagnetic energy emitted by partial discharges (PD) is another well-known application [8,9]. The relative position estimation amongst mobile objects or robots has also been studied [10]. All of these applications use or can use the techniques and methods presented in this paper to provide source localization.

There exist different techniques for indoor positioning, which also can be applied in outdoor localization. They can be classified into triangulation, received signal strength (RSS) and proximity [11,12]. The proximity algorithms are applied to mobile objects and rely upon a dense grid of antennas, which is not the case for the proposed study, where only four receivers are considered, and the emitter is in a fixed position. The RSS technique does not require time synchronization since it is based on a previous characterization of the area (or volume) where the emitter is found, which is made through a database of signal strengths at several positions as an initial step for localization. Afterwards, different localization algorithms based on pattern recognition techniques (such as k-nearest neighbour, artificial neural networks or support vector machines) compare the database, which helps to identify the RSS fingerprints with the actual emitter signal strength [12]. This is a time-consuming process, whose computational cost is high, as well. Moreover, in several applications (such as partial discharge localization in power systems), this previous RSS calibration for all the volume studied is not easy. Finally, triangulation algorithms show good results for unknown emitters, great flexibility in their application and are easy to implement in terms of computational burden and instrumentation, so they will be the topic of this paper.

Among the triangulation algorithms, there are several approaches [12,13]. One of them is a variation of the RSS algorithm (RSS indication), where the distance to the emitter is assessed through signal attenuation models. This approach is simple and cost-effective, but is highly affected by the absence of lines of sight between the emitter and receivers, diffraction, reflection, scattering, etc., which makes its application difficult in indoor settings. Another option is the localization through the angle of arrival (AoA, also called direction of arrival (DoA)), a technique that computes angles relative to multiple reference points; these angles define straight lines whose intersection defines the emitter position. The AoA requires using antenna arrays or highly directive antennas and large and complex hardware. In addition, this technique’s performance is performance degraded for long distances to the emitter, bad characterization of the antenna directivity and possible multipath propagation [13]. Finally, the use of the time of arrival and time differences of arrival between the emitter and the sensors has obtained good results. When the ignition time of the signal from the emitter is unknown, the emission attenuation cannot be accurately characterized and a reduced number of simple omnidirectional antennas (such as monopoles) is available, the use of TDoA is the most suitable technique, so this is why it has been selected for this study.

A main obstacle of the triangulation through TDoA is the need to solve a system of non-linear equations. The multilateration algorithms used to localize emitting sources can be classified into two categories: iterative and non-iterative methods. The iterative algorithms have a different logic in the search for the source solution. Standard least squares (SLS) and hyperbolic least squares (HLS) undertake the search using the Newton–Raphson optimization technique [14,15]. Other iterative algorithms base the search on metaheuristic techniques such as PSO [4,16,17]. The second category of non-iterative algorithms is widely used in GPS, where the emitters are orbiting satellites and the source target is to locate a sensor receiving the emissions. Many algorithms have been proposed to solve the set of non-linear equations. In [18,19], the system is reduced to linear equations representing them in another basis, and then, different techniques are applied to solve the system of equations. In this paper, we will focus the study on three commonly-used algorithms: the hyperbolic positioning algorithm (HPA) [20], the maximum likelihood estimator (MLE) [3] and the Bancroft algorithm [21].

Both iterative and non-iterative algorithms have advantages and disadvantages and do not always achieve the actual source position with confidence. This is because, in the first category, the algorithms do not always converge, and in the second category, the algorithms always yield two feasible solutions. The main disadvantages of iterative algorithms are that they require an initialization setup and a definition of the initial parameters by the user. This affects the convergence of the algorithms since the Newton–Raphson method does not provide convergence in some analysed points [22]. Consequently, it can provoke variations in the solution location accuracy and in the computational time. On the other hand, the non-iterative algorithms report directly two solutions, a positive and a negative root. In GPS, the root selection may be performed in different ways such as solving the clock error of a single receiver [23], using pseudo-ranges [21] and others. Commonly, in emitter localization, both solutions are feasible, though one root is closer than the other to the actual source solution, and it is not immediately obvious which determined root is the closest [24].

In this paper, a combined algorithm, MLE-HLS, is proposed to locate emitters solving the problem of determining the correct root. This algorithm is compared with other algorithms in terms of localization accuracy and computational time in relation to source accuracy and computational speed.

The paper is organized as follows: Section 2 provides an explanation and detailed formulation of all the algorithms used in the paper and presents the new combined MLE-HLS algorithm. The methodology to compare the algorithms is outlined in Section 3. The results of the comparison of the algorithms are presented in Section 4. Finally, conclusions and recommendations derived from the algorithm comparisons are summarised in Section 5.

## 2. Localization Techniques

**Figure 1:**
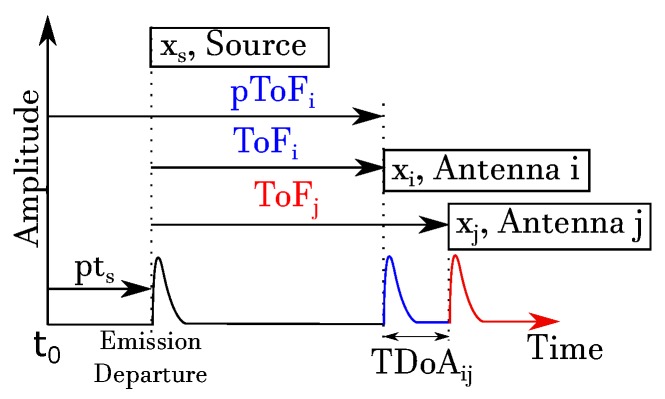
ToF, TDoA and pseudo-time of flight (pToF) representation for an emitting source $x_{s}$ and arbitrary receivers, e.g., sensors *i* and *j*.

For a better understanding of the algorithms, they have been divided into the two recognised categories: iterative and non-iterative. Every multilateration algorithm has a different formulation, but all of them are based on the fact that the distance travelled by the emissions is equal to the time spent in flight multiplied by the speed propagation in the medium under consideration, free space, oil, water, etc. Though the methods can be applied to any emitting source, this paper is, henceforth, based on the use of antennas as sensors to localize partial discharges in the UHF range, so the medium would be free space. The algorithms use different time variables, which are described below. The ToF of the source emission represents the time spent by the emission to travel from the emitter to the receiver. In some applications, it is difficult to know precisely the time when the emission departs. In such cases, it is preferable to use the TDoA from the emitter to a pair of receivers and then determine the source location with a system of non-linear equations. The pToF is an alternative time measure, which represents the ToF of the emission to any sensor with a fixed offset included, $pt_{s}$. This time offset represents the time elapsed between the start of a specified clock, $t_{0}$, and the emission departure. Figure 1 represents the above described time variables. Determining the onset of the pulse to obtain these times is arguably the most critical task when localizing the source with multilateration techniques [25]. Unfortunately, the detected pulses are usually small with low signal-to-noise ratios, easily leading to errors in the calculation of their onset. The effect of almost negligible errors in the onset can shift the calculated position of the source several meters away when measuring signals in the UHF range. Therefore, robust picking algorithms have to be devised to minimize the error in the time variables. Nevertheless, though this type of error is not in the scope of this work, it could be considered as an additional source of uncertainty in the inputs to the algorithms compared in this paper together with the digitizing errors explained in Section 3.

The spatial relationship between the ToF, $t_{i}$, of the emission from the source to the *i*-th receiver is:

(1) $$t_{i}\cdot c=D_{i}$$

where *c* is the propagation speed equal to $3\times 10^{8}\;{\mathrm{ms}}^{-1}$ in free space and $D_{i}$ represents the distance from the position of the *i*-th antenna $P_{i}=\left(x_{i},y_{i},z_{i}\right)$ to the source position $P_{s}=\left(x_{s},y_{s},z_{s}\right)$ in three dimensions, being calculated through:

(2) $$D_{i}=\sqrt{{\left(x_{s}-x_{i}\right)}^{2}+{\left(y_{s}-y_{i}\right)}^{2}+{\left(z_{s}-z_{i}\right)}^{2}}$$

The TDoF between signals received at the *i*-th and *j*-th, $t_{ij}$, antennas, is related to the spatial variables through:

(3) $$t_{ij}\cdot c=D_{i}-D_{j}$$

In the case of pToF, the relationship is similar to the ToF situation, but considers the time offset. The pToF for the *i*-th antenna is denoted as $t_{i}^{\prime }$ where $t_{i}^{\prime }=t_{i}+t_{s}$, and $t_{s}$ is the time offset elapsed between the instant when any time acquisition starts, t=0, and the instant at which the source emission departs.

The minimum number of antennas to localize the source in three dimensions is four, which is the most common application to locate the source of partial discharges in power systems. These four receivers need to be properly deployed with at least one receiver allocated in a different plane than the other three receivers [25]. Increasing the number of antennas would pose both hardware and budgetary problems since high speed digitizers have a maximum of four channels and, in any other acquisition system, every new channel dramatically increases the cost of the equipment.

### 2.1. Iterative Algorithms

#### 2.1.1. Standard Least Squares

The SLS algorithm estimates the emitter position $P_{s}$, i.e., ${\hat{P}}_{s}=\left({\hat{x}}_{s},{\hat{y}}_{s},{\hat{z}}_{s}\right)$ minimizing the OF defined by Equation (4) using Newton–Raphson iteration [14],

(4) $$OF\left({\hat{x}}_{s},{\hat{y}}_{s},{\hat{z}}_{s},{\hat{t}}_{1}\right)=\sum_{i=1}^{N}{\left({\hat{D}}_{i}-{\hat{t}}_{i}\cdot c\right)}^{2}$$

where *N* is the number of antennas. Since $t_{i}$ cannot be measured because the emission departure instant is unknown, it is estimated through ${\hat{t}}_{i}=\left({\hat{t}}_{1}+t_{i1}\right)$ where ${\hat{t}}_{1}$ is the estimated ToF of the source emission to the reference antenna, i.e., approaching Antenna 1. The TDoA between Antenna 1 and the *i*-th antenna is represented by $t_{i1}$, with $t_{11}=0$.

Minimization is achieved by applying the variable partial derivatives to Equation (4), i.e., $\left({\hat{x}}_{s},{\hat{y}}_{s},{\hat{z}}_{s},{\hat{t}}_{1}\right)$ and setting equal to zero; see Equation (5).

(5) $$\frac{\partial OF}{\partial {\hat{x}}_{s}}=0,\;\frac{\partial OF}{\partial {\hat{y}}_{s}}=0,\;\frac{\partial OF}{\partial {\hat{z}}_{s}}=0,\;\frac{\partial OF}{\partial {\hat{t}}_{1}}=0$$

With respect to ${\hat{x}}_{s}$, the minimisation Equation (6) is obtained:

(6) $$\frac{\partial OF}{\partial {\hat{x}}_{s}}={\hat{x}}_{s}-\frac{1}{N}\sum_{i=1}^{N}\left(x_{i}+\frac{\left({\hat{x}}_{s}-x_{i}\right)\left({\hat{t}}_{1}+t_{i1}\right)c}{{\hat{D}}_{i}}\right)=0$$

As this algorithm is iterative and searches for convergence, it can be assumed that ${\hat{x}}_{s}\left(l\right)≃{\hat{x}}_{s}\left(l-1\right)$. Then, Equation (6) can be modified to Equation (7) where the estimated source coordinate ${\hat{x}}_{s}$ in the *l* iteration, ${\hat{x}}_{s}\left(l\right)$, is defined as a function of the value ${\hat{x}}_{s}$ in the previous iteration (l−1), denoted as ${\hat{x}}_{s}\left(l-1\right)$.

(7) $${\hat{x}}_{s}\left(l\right)=\frac{1}{N}\sum_{i=1}^{N}\left(x_{i}+\frac{\left({\hat{x}}_{s}\left(l-1\right)-x_{i}\right)\left({\hat{t}}_{1}\left(l-1\right)+t_{i1}\right)c}{{\hat{D}}_{i}\left(l-1\right)}\right)$$

Equation (8) may be obtained through performing the same minimisation operation for all source coordinates $\left({\hat{x}}_{s},{\hat{y}}_{s},{\hat{z}}_{s}\right)$ and grouping them into the unique vector ${\hat{P}}_{s}\left(l\right)$ representing the estimated source position of the source.

(8) $${\hat{P}}_{s}\left(l\right)=\frac{1}{N}\sum_{i=1}^{N}\left(P_{i}+\frac{\left({\hat{P}}_{s}\left(l-1\right)-P_{i}\right)\left({\hat{t}}_{1}\left(l-1\right)+t_{i1}\right)c}{{\hat{D}}_{i}\left(l-1\right)}\right)$$

The estimated ToF from the source to Antenna 1, ${\hat{t}}_{1}$, is obtained as a function of ${\hat{P}}_{s}\left(l-1\right)$ embedded in ${\hat{D}}_{i}\left(l-1\right)$, from the time-related partial derivative in Equation (5):

(9) $${\hat{t}}_{1}\left(l\right)=\frac{1}{N}\sum_{i=1}^{N}\left(\frac{{\hat{D}}_{i}\left(l-1\right)}{c}-t_{i1}\right)$$

In the event that the algorithm converges before reaching the required maximum iterations *L*, the algorithm stops. The convergence criterion is defined by Equation (10).

(10) $$\begin{array}{c} {\hat{x}}_{s}\left(l\right)-{\hat{x}}_{s}\left(l-1\right)<\epsilon_{d}\\ {\hat{y}}_{s}\left(l\right)-{\hat{y}}_{s}\left(l-1\right)<\epsilon_{d}\\ {\hat{z}}_{s}\left(l\right)-{\hat{z}}_{s}\left(l-1\right)<\epsilon_{d}\\ {\hat{t}}_{1}\left(l\right)-{\hat{t}}_{1}\left(l-1\right)<\epsilon_{t} \end{array}$$

In the simulations carried out in this paper, the maximum iteration number, *L*, is fixed to $10^{7}$, the distance error bound, $\epsilon_{d}$, is set to $10^{-13}$ m, the time error bound $\epsilon_{t}$ to $10^{-13}$ s and the localization start point is defined as ${\hat{P}}_{s}\left(0\right)=\left(0,0,0\right)$ and ${\hat{t}}_{1}\left(0\right)=0$. These conditions are defined in an attempt to find the exact solution, although this implies high computational time, as demonstrated in [26].

#### 2.1.2. Hyperbolic Least Squares

In this technique, the OF is similar to Equation (4), but the difference is in the time variable, which in this case is the TDoA, providing representations of the possible source solutions of the equations as hyperbolas. The OF for HLS, Equation (11), can be obtained by applying Equation (3) for the reference antenna to the other three antennas and adding all of them together, e.g., [15].

(11) $$OF\left({\hat{x}}_{s},{\hat{y}}_{s},{\hat{z}}_{s}\right)=\sum_{i=2}^{N}{\left({\hat{D}}_{i}-{\hat{D}}_{1}-t_{i1}c\right)}^{2}$$

The HLS iterative algorithm also bases the calculation of the source position on Newton–Raphson iteration. Again, the initial procedure is to calculate and set equal to zero the partial derivatives of the OF, i.e., Equation (11), with respect to $\left({\hat{x}}_{s},{\hat{y}}_{s},{\hat{z}}_{s}\right)$ as shown in Equation (12).

(12) $$\frac{\partial OF}{\partial {\hat{x}}_{s}}=0,\;\frac{\partial OF}{\partial {\hat{y}}_{s}}=0,\;\frac{\partial OF}{\partial {\hat{z}}_{s}}=0$$

Operating and solving Equation (12) in the same way as in Section 2.1.1, three equations are obtained, which can be expressed as before through grouping $\left({\hat{x}}_{s},{\hat{y}}_{s},{\hat{z}}_{s}\right)$ in ${\hat{P}}_{s}$:

(13) $$\begin{array}{cc} {\hat{P}}_{s}\left(l\right) & =\frac{1}{2(N-1)}\sum_{i=2}^{N}(P_{i}+P_{1}\\ & \;+\left({\hat{P}}_{s}\left(l-1\right)-P_{i}\right)\left(\frac{{\hat{D}}_{1}+t_{i1}c}{{\hat{D}}_{i}}\right)\\ & \;+\left({\hat{P}}_{s}\left(l-1\right)-P_{1}\right)\left(\frac{{\hat{D}}_{i}-t_{i1}c}{{\hat{D}}_{1}}\right)) \end{array}$$

Defining the initial value ${\hat{P}}_{s}\left(0\right)=\left(0,0,0\right)$, Equation (13) allows the source position to be found through iteration. The solution represents the intersection of the hyperbolas with foci defined by the antennas’ position and the measured TDoAs. The initial conditions and the constraints of the simulations are the same as for SLS. The convergence criterion is defined by the first three equations in Equation (10).

#### 2.1.3. Particle Swarm Optimization Based on HLS

PSO is a metaheuristic technique that searches the optimum inspired by the way birds or fish look for food. “Particles” are deployed in the solution space with each particle having three variables $\left(x_{s},y_{s},z_{s}\right)$. *K* moving particles change their position $P_{k}$ in each iteration through:

(14) $$P_{k}\left(l\right)=P_{k}\left(l-1\right)+v_{k}\left(l\right)$$

The displacement or velocity of the *k*-th particle in the *l*-iteration, $v_{k}\left(l\right)$, considers the particle velocity in the previous iteration, $v_{k}\left(l-1\right)$, its personal best position, $P_{k,\mathrm{best}}\left(l-1\right)$, and the global best position of the swarm $P_{\mathrm{best}}\left(l-1\right)$, found at any time during the search. $v_{k}\left(l\right)$ is derived through:

(15) $$\begin{array}{cc} v_{k}\left(l\right) & =\omega v_{k}\left(l-1\right)\\ & \;+C_{1}U_{1}\left(P_{k,\mathrm{best}}\left(l-1\right)-P_{k}\left(l-1\right)\right)\\ & \;+C_{2}U_{2}\left(P_{\mathrm{best}}\left(l-1\right)-P_{k}\left(l-1\right)\right) \end{array}$$

where:

(16) $$v_{k}\left(l\right)=\left[\begin{array}{c} v_{k,x}\left(l\right)\\ v_{k,y}\left(l\right)\\ v_{k,z}\left(l\right) \end{array}\right],P_{k}\left(l\right)=\left[\begin{array}{c} x_{k}\left(l\right)\\ y_{k}\left(l\right)\\ z_{k}\left(l\right) \end{array}\right]$$

where k=1,2,...,K and l=1,2,...,L. The particle inertia, ω, varies from 0.9 in the first iteration to 0.4 in the *L*-th iteration, e.g., [27]. This induces high movement of the particles at the beginning of the simulation to explore large source regions and focus the search around the optimum location, then moving the particles slowly at the end of the simulation. $C_{1}$ and $C_{2}$ allow balance to the influence of the personal best or global best position in the search. $U_{1}$ and $U_{2}$ are line matrices with three elements randomly distributed between zero and one, which randomizes the movement of the particles, and are generated in each iteration to introduce randomness in the search.

Every particle is evaluated in the minimization OF, Equation (17), based on HLS of Equation (11). It considers six TDoAs when four antennas are used in order to obtain better accuracy in the localization.

(17) $$OF\left({\hat{x}}_{s},{\hat{y}}_{s},{\hat{z}}_{s}\right)=\sum_{i=1}^{N-1}\sum_{j=i+1}^{N}{\left({\hat{D}}_{i}-{\hat{D}}_{j}-t_{ij}c\right)}^{2}$$

The specific algorithm steps are as follows:

1. *K* particles are spread in the space of solutions. The initial velocity is set to zero for all particles.
2. Each particle position is evaluated using Equation (17).
3. If some particle improves its local best position, it is updated. If some particle improves the global best position, it is also updated.
4. The velocity Equation (15) and the position Equation (14) are updated for each particle in the swarm.
5. If the maximum number of iterations *L* is reached or when all the particles are located close to the same point, the algorithm ends, and the solution is the position of $P_{\mathrm{best}}\left(L\right)$. Otherwise, go to Step 2.

### 2.2. Non-Iterative Algorithms

#### 2.2.1. Hyperbolic Positioning Algorithm

The HPA algorithm was developed by Ralph Bucher for GPS location using four fixed stations [20] and can be applied to any emitter localization. This algorithm uses the TDoA as HLS in Section 2.1.2.

The position of the source is defined as the intersection of four hyperbolas based on Equation (3):

(18) $$\begin{array}{cc} D_{12}=D_{1}-D_{2} & =t_{12}c\\ D_{13}=D_{1}-D_{3} & =t_{13}c\\ D_{32}=D_{3}-D_{2} & =t_{32}c\\ D_{34}=D_{3}-D_{4} & =t_{34}c \end{array}$$

These equations are solved using several intermediate variables with the final target of obtaining the source coordinates. The full formulation is developed in detail in [20]. As a result of the equation system, the coordinates of the position of the source $\left(x_{s},y_{s},z_{s}\right)$ are defined through equations that report two direct solutions, i.e., a positive root and a negative root.

#### 2.2.2. Bancroft Algorithm

The Bancroft algorithm was developed by Stephen Bancroft also for GPS location [21]. This algorithm uses a different equation system to find the source position through the Lorenz inner product for four space arrays. The four space array for the antennas and the source is defined through Equation (19),

(19) $$a_{i}=\left[\begin{array}{c} x_{i}\\ y_{i}\\ z_{i}\\ c\cdot pt_{i} \end{array}\right],\;a_{s}=\left[\begin{array}{c} x_{s}\\ y_{s}\\ z_{s}\\ c\cdot pt_{s} \end{array}\right]$$

where $pt_{i}$ denotes the pToF measurements taken from each of the four antennas. In radio frequency (RF) emitter localization, $pt_{i}$ is the absolute ToF at the *i*-th antenna plus an additional offset $pt_{s}$. The source position is defined by $\left(x_{s},y_{s},z_{s}\right)$, and $pt_{s}$ is the elapsed time between the instant when the receiver acquisitions starts and the instant when the emission departs. The Lorenz inner product of $a_{i}$ and $a_{s}$ can be calculated through:

(20) $$\langle a_{i},a_{s}\rangle =x_{i}x_{s}+y_{i}y_{s}+z_{i}z_{s}-c^{2}\cdot pt_{i}\cdot pt_{s}$$

The Bancroft approach pivots around the variables defined above. The source position can be obtained following the operations detailed below. Defining:

(21) $$A={\left(a_{1}\;,a_{2}\;,a_{3}\;,a_{4}\right)}^{T}=\left[\begin{array}{cccc} x_{1} & y_{1} & z_{1} & -c\cdot pt_{1}\\ x_{2} & y_{2} & z_{2} & -c\cdot pt_{2}\\ x_{3} & y_{3} & z_{3} & -c\cdot pt_{3}\\ x_{4} & y_{4} & z_{4} & -c\cdot pt_{4} \end{array}\right]$$

then computation of the four space u and v vectors takes place:

(22) $$\begin{array}{cc} u & =A^{-1}i_{0}\\ v & =A^{-1}r \end{array}$$

where:

(23) $$i_{0}=\left[\begin{array}{c} 1\\ 1\\ 1\\ 1 \end{array}\right],r=\frac{1}{2}\left[\begin{array}{c} \langle a_{1},a_{1}\rangle \\ \langle a_{2},a_{2}\rangle \\ \langle a_{3},a_{3}\rangle \\ \langle a_{4},a_{4}\rangle \end{array}\right]$$

Scalar coefficients *E*, *F* and *G* are then calculated in Equations (24).

(24) $$\begin{array}{c} E=\langle u,u\rangle \\ F=\langle u,v\rangle -1\\ G=\langle v,v\rangle \end{array}$$

The final Equation (25) is defined as:

(25) $$E\lambda^{2}+2F\lambda +G=0$$

Solving Equation (25), two solutions, $\lambda^{+}$, the positive root, and $\lambda^{-}$, the negative root, are obtained. Two possible source positions are then calculated using Equation (26).

(26) $${a_{s}}^{+}=\lambda^{+}u+v=\left[\begin{array}{c} x_{s}^{+}\\ y_{s}^{+}\\ z_{s}^{+}\\ c\cdot pt_{s}^{+} \end{array}\right],\;{a_{s}}^{-}=\lambda^{-}u+v=\left[\begin{array}{c} x_{s}^{-}\\ y_{s}^{-}\\ z_{s}^{-}\\ c\cdot pt_{s}^{-} \end{array}\right]$$

In GPS applications, the criterion to choose the correct root is based on clock synchronisation because only one solution fits. In source location, this criterion cannot be applied because the time when the emission departs is unknown, so both solutions would be, in principle, valid in the analysis.

#### 2.2.3. Maximum Likelihood Estimator Algorithm

The MLE algorithm was developed by Chan and Ho for GPS location and also reports two explicit solutions [3]. Equations (1) and (2) are the initial equations, which combined produce:

(27) $$D_{i}^{2}=x_{s}^{2}+y_{s}^{2}+z_{s}^{2}-2x_{s}x_{i}-2y_{s}y_{i}-2z_{s}z_{i}+K_{i}$$

where:

(28) $$K_{i}=x_{i}^{2}+y_{i}^{2}+z_{i}^{2}$$

Considering Antenna 1 as the reference, the TDoA for each pair of antennas can be represented by $t_{i1}$ with i={2,3,4} representing the non-reference antennas. Using the two first terms of hyperbolic Equation (3), then Equation (29) is obtained:

(29) $$D_{i}=D_{i1}+D_{1}$$

Substituting (29) in (27) results in:

(30) $$\begin{array}{c} D_{i1}^{2}+2D_{i1}D_{1}+D_{1}^{2}\\ \;=x_{s}^{2}+y_{s}^{2}+z_{s}^{2}-2x_{s}x_{i}-2y_{s}y_{i}-2z_{s}z_{i}+K_{i} \end{array}$$

Evaluating (30) minus (27) considering i=1, then:

(31) $$\begin{array}{c} D_{i1}^{2}+2D_{i1}D_{1}=-2x_{s}\left(x_{i}-x_{1}\right)\\ \;-2y_{s}\left(y_{i}-y_{1}\right)-2z_{s}\left(z_{i}-z_{1}\right)+K_{i}-K_{1} \end{array}$$

Equation (31) can be expressed in matrix form as follows:

(32) $$\begin{array}{cc} \left[\begin{array}{c} x_{s}\\ y_{s}\\ z_{s} \end{array}\right] & =-{\left[\begin{array}{ccc} x_{2}-x_{1} & y_{2}-y_{1} & z_{2}-z_{1}\\ x_{3}-x_{1} & y_{3}-y_{1} & z_{3}-z_{1}\\ x_{4}-x_{1} & y_{4}-y_{1} & z_{4}-z_{1} \end{array}\right]}^{-1}\\ & \times \left\{\left[\begin{array}{c} D_{21}\\ D_{31}\\ D_{41} \end{array}\right]D_{1}+\frac{1}{2}\left[\begin{array}{c} D_{21}^{2}-K_{2}+K_{1}\\ D_{31}^{2}-K_{3}+K_{1}\\ D_{41}^{2}-K_{4}+K_{1} \end{array}\right]\right\} \end{array}$$

There are three equations and four unknown variables $x_{s},y_{s},z_{s},D_{1}$. The fourth equation to help solve the system is found in Equation (27). Substituting $\left(x_{s},y_{s},z_{s}\right)$ from Equation (32) in Equation (27) with i=1, a quadratic result in $D_{1}$ is obtained. Substituting the positive and negative roots of $D_{1}$ back into Equation (32), two possible source positions are provided, i.e., $\left(x_{s}^{+},y_{s}^{+},z_{s}^{+}\right)$, a positive root, and $\left(x_{s}^{-},y_{s}^{-},z_{s}^{-}\right)$, a negative root. However, as before, the correct solution is not clearly known.

#### 2.2.4. Combined MLE-HLS Algorithm

A combined algorithm based on the MLE algorithm outlined in Section 2.2.3 and the HLS algorithm outlined in Section 2.1.2 was previously proposed in [28]. The MLE algorithm reports two feasible solutions, with one of them closer to the actual source position than the other. However, the desired solution is not always located in the position given by the positive root. With MLE-HLS, the correct solution selection is carried out using the OF based on HLS, Equation (17).

The rationale for combining HLS and MLE is as follows. The HLS OF is based on using all available information from the system, i.e., all TDoAs, as well as all receiver spatial distances. As two solutions of MLE will exist, the solution that explicitly produces the lowest OF value is intuitively the preferred solution since it provides minimal deviation for all possible system spatial setup parameter measurements and relative time calculation determinations. It may also be noted that the HLS OF, Equation (17), has been shown to provide good location accuracy when employed within the PSO algorithm, thus demonstrating its ability as a reasonable metric for location determinations.

The performance of any source location algorithm depends on the software configuration. It is possible to report results with high resolution accuracy, e.g., micrometers, but, there is generally no need to locate the sources with such accuracy in most applications. Thus, in the presented simulations, the solutions are rounded to millimetres before evaluating Equation (17).

## 3. Methodology

The inputs to the localization algorithms are the receiver antenna positions and the time variables ToF, TDoA or pToF depending on the algorithm. As the purpose of the paper is to evaluate algorithm performance through simulations, the time variables are initially calculated from the spatial geometry rather than from time measurements. The source position therefore has to be previously defined to carry out the calculation of the time variables. Then, the ToF is defined as the distance between the source and the respective antenna and divided by the speed of propagation, *c*. The TDoA between two antennas is the subtraction between their ToF. The pToF is calculated adding an offset to the ToF variables.

The simulation procedure has four steps. The first one is to set the four antennas and the source position. The second one is to calculate the exact theoretical ToF, TDoA and pToF for that geometric configuration. The third step is using these times to calculate back the source position for every algorithm using the antenna positions and the time variables. The fourth step is to calculate the computational time spent in processing the location and to determine the errors in source location for every algorithm.

The procedure described above is performed for two situations; firstly, under precise theoretical values for the time variables and, secondly, introducing a digitizing sampling error on the time measurements, which reflects a more practical application. Under theoretical conditions, the time variables have high decimal value time resolution. We have introduced digitizing errors in the theoretic values of ToF, TDoA and pToF, which would give an idea of what would be the behaviour of the algorithms when these times have other types of uncertainties such as when the environment is full of metallic structures. To emulate the time sampled digitizing error, the time variables are rounded to the nearest time sample. For evaluation purposes, in this paper, an acquisition system with sampling frequency $f_{s}$ = 10 GS/s is presumed. The time interval between samples is then $T_{s}=1/f_{s}=0.1$ nanoseconds. When multiplied by the speed of the light, *c*, this results in a maximum location error of 3 cm for each time measurement. In this case, if the TDoA between two antennas multiplied by *c* is 1 m, digitalizing the discrete TDoA, with a 3-cm error, will result in an overall location error of 0.99 m or 1.02 m. However, suitable interpolation between time sampled points can reduce the location error magnitude [29]. In the previous example, interpolating with 10 samples between time points, the effective sampling frequency may be increased to $f_{s}$ = 100 GS/s, and $T_{s}$ is effectively reduced to 0.01 ns, resulting in an location error of around 3 mm (see Section 4.2).

In some cases, when the non-iterative algorithms are executed under digitizing time errors, the localization can yield a solution with real and imaginary components. This is caused because the time round up introduces an error in the process, which provokes square roots of negative values. In these cases, the imaginary term is omitted because the solution space is the real 3D space. In the iterative algorithms, this problem does not exist because the search is always performed in real 3D space.

The simulations were carried out in a computer with an Intel(R) Core(TM) i7-3630QM CPU @ 2.40 GHz processor, with RAM memory of 8.00 GB (7.89 GB usable) using MATLAB Version R2016b. The priority of the MATLAB process in the computer was set to real time.

The source localization determinations are converted from Cartesian coordinates to spherical coordinates, i.e., ${\hat{P}}_{s}=\left(\hat{r},\hat{\theta },\hat{\varphi }\right)$. This procedure permits distinguishing errors in distance estimation $\left(\hat{r}\right)$ and errors in angular direction estimation $\left(\hat{\theta },\hat{\varphi }\right)$. The errors are calculated from the estimated source position ${\hat{P}}_{s}$ to the actual source position $P_{s}=\left(r,\theta ,\varphi \right)$ through:

(33) $$\begin{array}{cc} \Delta \hat{r} & =|\hat{r}-r|\\ \Delta \hat{\theta } & =|\hat{\theta }-\theta |\\ \Delta \hat{\varphi } & =|\hat{\varphi }-\varphi | \end{array}$$

**Figure 2:**
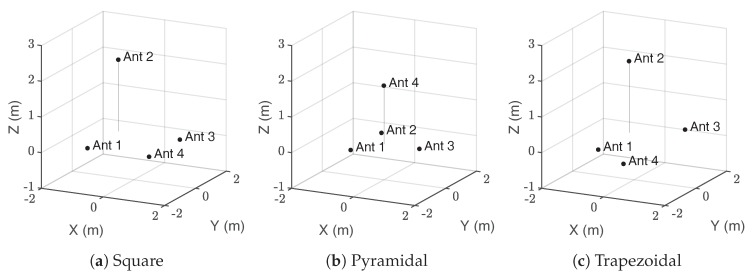
Representation of the positions of the antennas represented as black dots, in a Cartesian coordinate system. Ant stands for antenna.

The relative position of the antennas with respect to the source plays an important role in its accurate localization. When there are errors in the determination of the exact value of the TDoA, the resulting positions of the source are scattered around the actual position. However, it has been proven in [25] that there are certain bearings for which the standard deviation of the positions is lower than for other directions. Therefore, the accuracy in those bearings, dependent on the antenna layout, would be larger. In order to enable a more robust evaluation of the algorithms, the simulations considering digitizing errors and exact values in the TDoA are conducted for three widely-used antenna layouts (square, pyramid and trapezoidal) in the localization of partial discharge sources; see Figure 2. Table 1 shows the detailed coordinates of each antenna array configuration used in the simulation studies.

**Figure 3:**
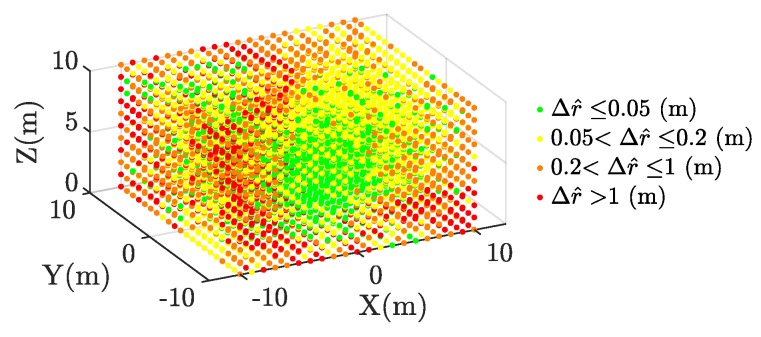
Example of source emitter radius error $\Delta \hat{r}$ analysed with MLE-hyperbolic least squares (HLS) under time sampled digitizing error conditions.

A wide range of source positions is simulated in a geometrical space enclosing the antenna layouts. Source points are located every meter in each direction inside a volume 20×20×10 m centred at (0,0,5) m. Figure 3 shows an example of all the possible positions of the source in this volume. It is possible to visualise the accuracy in the estimated source location through color grading. For example, using MLE-HLS under some time sampling constraint for the trapezoidal antennas configuration, the estimated radius position error in $\hat{r}$ may be visualized as in Figure 2c. With green representing a more accurate determination and red indicating a larger source error, it can be seen that as long as the source is close to the antenna array, the accuracy in $\hat{r}$ is high.

## 4. Results

In this section, all the results obtained from the simulations are generated following the procedure described in Section 3.

### 4.1. Theoretical Conditions

The results of the simulations presented in this section are undertaken for theoretical conditions. This means that the ToF, TDoA and pToF used in the location algorithms are the theoretical values with high time resolution. The aim of these simulations is to test the performance of the algorithms when finding a solution to the relevant OF.

#### 4.1.1. Accuracy

**Figure 4:**
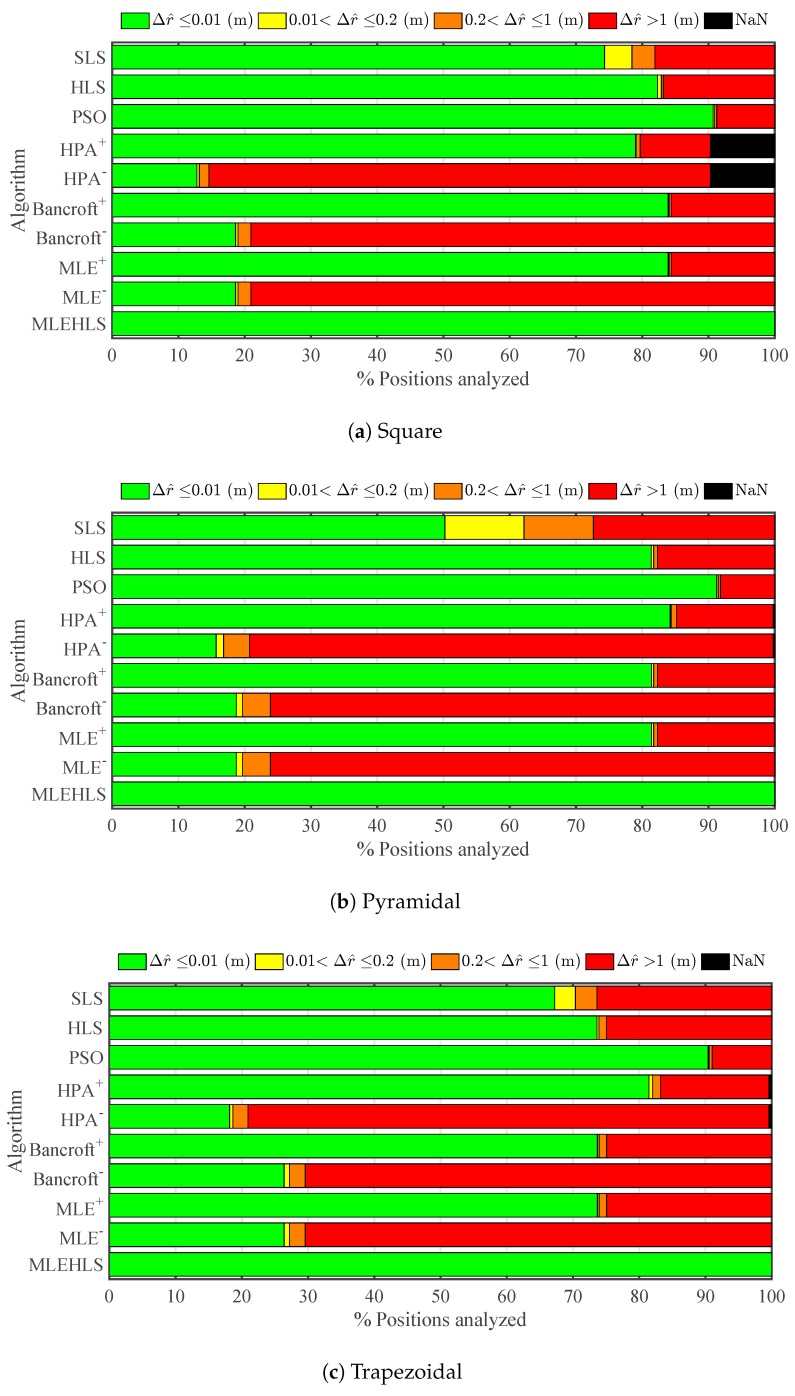
Percentage of positions located within the errors in the radius $\Delta \hat{r}$ defined in the legend under theoretical conditions for each algorithm and antenna array configuration.

Every algorithm is evaluated for the same source positions detailed in Section 3; see Figure 3. The source location accuracy in radius, elevation and azimuth for all methods has been quantified for all volume positions for the three antenna configurations. Figure 4 shows the percentage of points located within the radius errors defined by the legend for every algorithm in each antenna configuration. Table 2 displays the percentage number of positions located with less than 1-cm accuracy in the radius calculations. It may be seen that the best algorithm is MLE-HLS, yielding a 100% accuracy of points less than 1 cm for every antenna layout. The next best algorithm is PSO, which localizes 90.8% of points with an error lower than 1 cm. The positive root of the other non-iterative algorithms localizes 79–80% of the points with good accuracy. On the other hand, the negative root only produces an accuracy of 15–21%.

**Figure 5:**
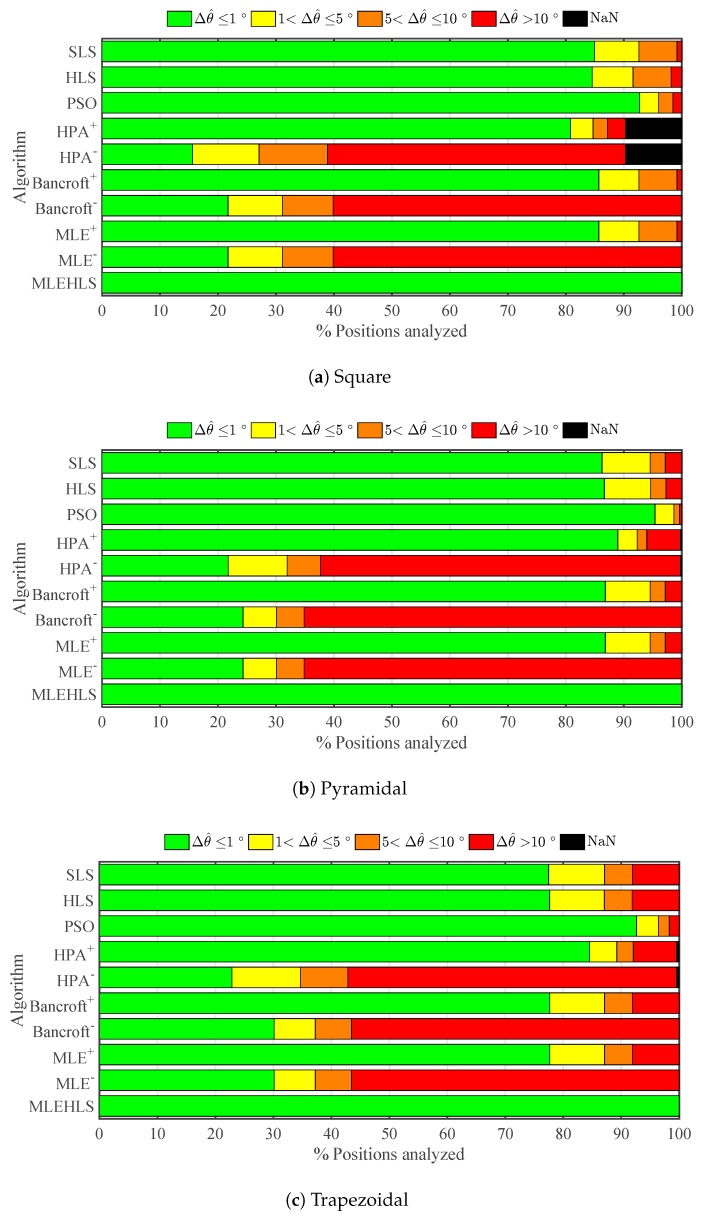
Percentage of positions located within the errors in the elevation angle $\Delta \hat{\theta }$ in degrees defined in the legend under theoretical conditions for each algorithm and antenna array configuration.

The percentage number of points located within a range of errors in elevation angle for each algorithm and every antenna array is shown in Figure 5. The percentage number of positions located with an elevation angle error lower than $1^{\circ }$ is highlighted in Table 3. In summary, the best algorithm is again MLE-HLS, which reports 100% of the analysed positions having an elevation angle error less than $1^{\circ }$ in every antenna layout. The second best algorithm is again PSO, which locates 93.6% of the positions with high accuracy. The other algorithms are not as good, locating with high accuracy only 45–88% of the points. The negative roots of the non-iterative algorithms report very poor results, locating with high accuracy a mere 20–25% of the positions.

**Figure 6:**
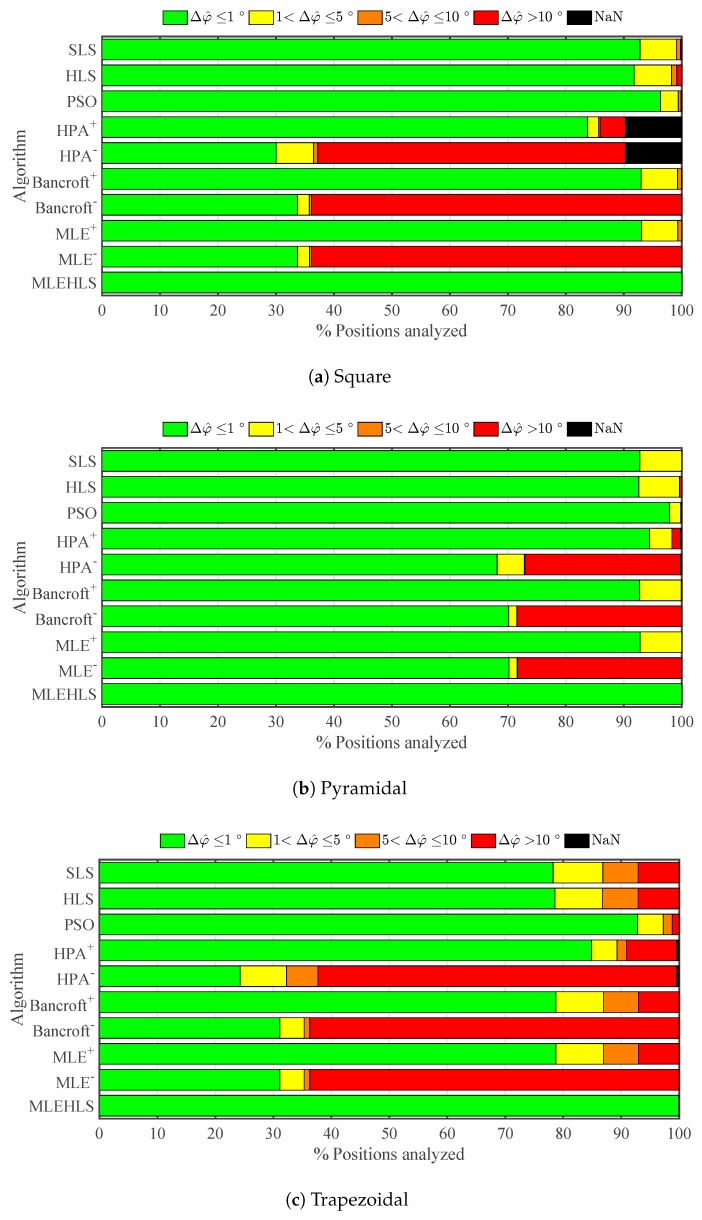
Percentage of positions located within the errors in the azimuth angle $\Delta \hat{\varphi }$ in degrees defined in the legend under theoretical conditions for each algorithm and antenna array configuration.

The same calculations are performed for azimuth angle, and the results are shown in Figure 6 and in Table 4. The best algorithm is again MLE-HLS with 100% of positions located with an azimuth angle error lower than $1^{\circ }$. PSO is again the second best with the other algorithms performing less well. In general, azimuth angle is determined with better accuracy for more positions than elevation angle.

#### 4.1.2. Computational Time

**Figure 7:**
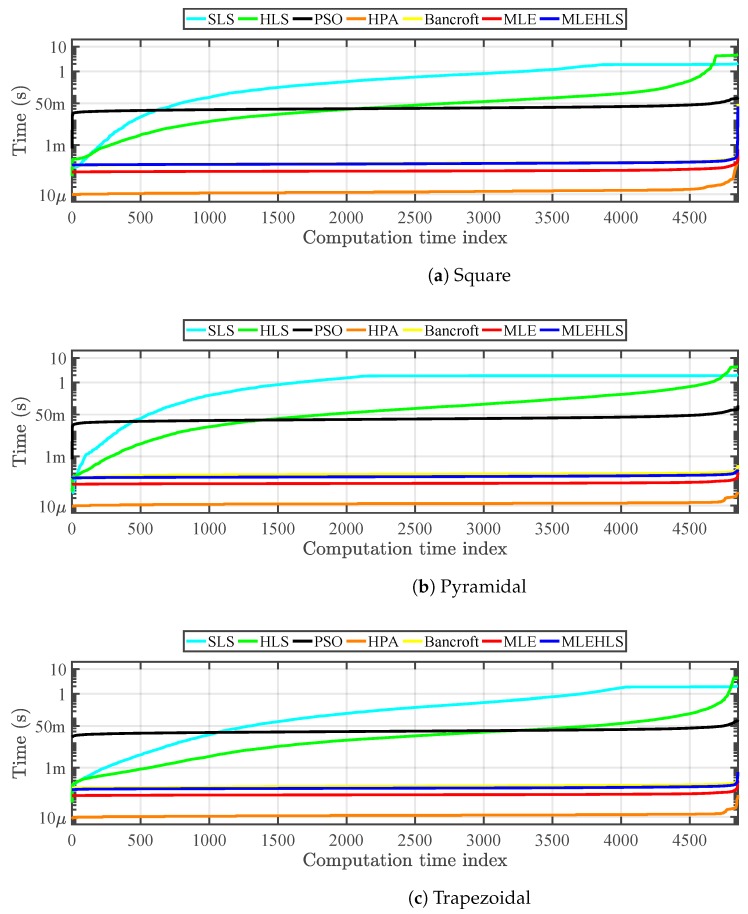
Computational time spent for each position analysed in theoretical conditions by each algorithm.

The computational time for each position, algorithm and antenna layout is shown in Figure 7. The vertical axis is the time required by the algorithm to reach a solution in logarithmic scale sorted from the lowest to the highest times. The horizontal axis shows all possible positions of the source according to the volume shown in Figure 3. Since the computation times are sorted, the indices in the horizontal axis start in the positions where it is easier to find a solution and end with the positions where it is harder.

It can be seen that the HPA algorithm is the fastest, needing only 13 μs to localize one position. MLE, MLE-HLS and Bancroft are also fast requiring 84–190 μs. As expected, the iterative algorithms are slower than the non-iterative algorithms, requiring from 33 ms–890 ms.

Most of the algorithms have quite a constant computational time except SLS and HLS, which display high computational differences due to the fact that the number of iterations needed in each source position is not the same. Indeed, the initial position in the search space is set equal to (0,0,0), so the algorithms have to translate the solutions closer to the actual position until convergence is reached. When the source is placed near the initial position, the translation is shorter and the algorithms require less iterations and, therefore, less time to converge compared to more distant source locations. Table 5 highlights the mean computational time for one source location for each algorithm for every antenna configuration.

### 4.2. Time Sampling Digitizing Error

The results of the simulations presented in this section are carried out considering sampled digitizing error on the time variables as explained in Section 3. Time sampled interpolation has also been included.

#### 4.2.1. Accuracy

**Figure 8:**
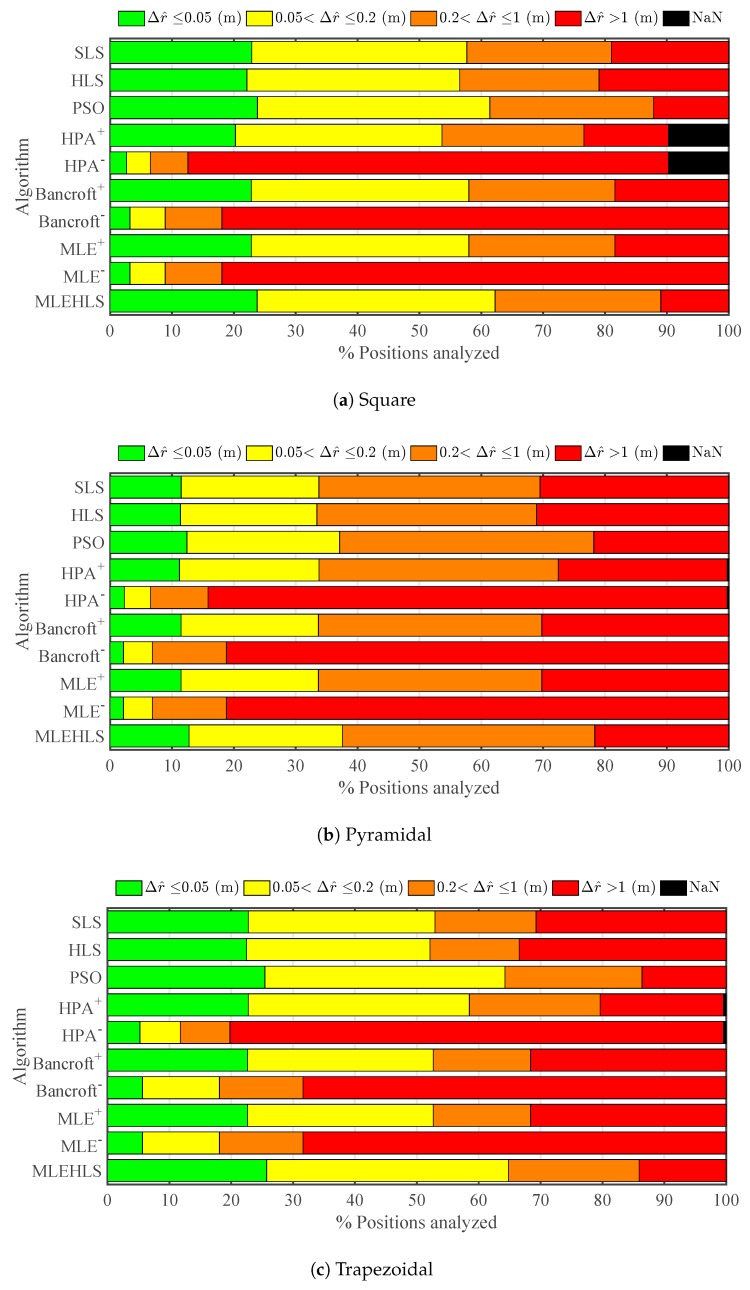
Percentage of positions located within the errors in the radius $\Delta \hat{r}$ defined in the legend under digitizing error in time variables for each algorithm and antenna array configuration.

**Figure 9:**
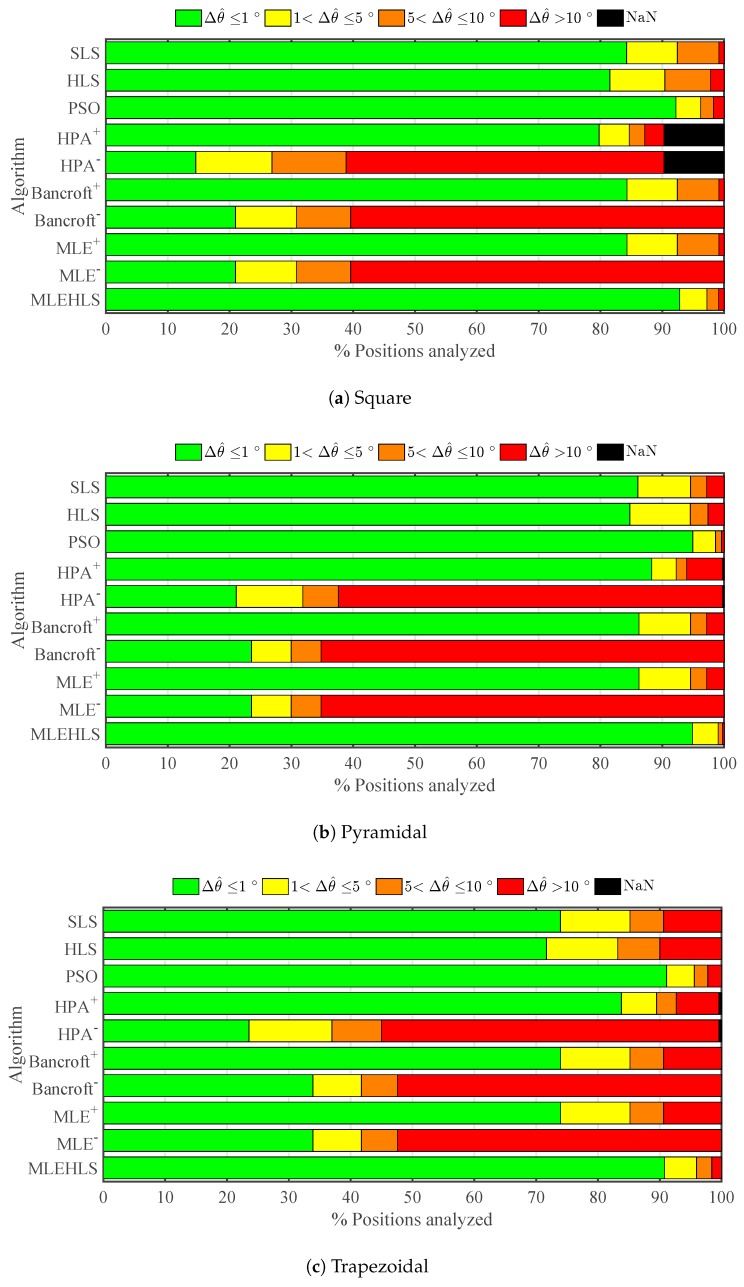
Percentage of positions located within errors bands in the elevation angle $\Delta \hat{\theta }$ under time sampled digitizing error for each algorithm and antenna array configuration.

**Figure 10:**
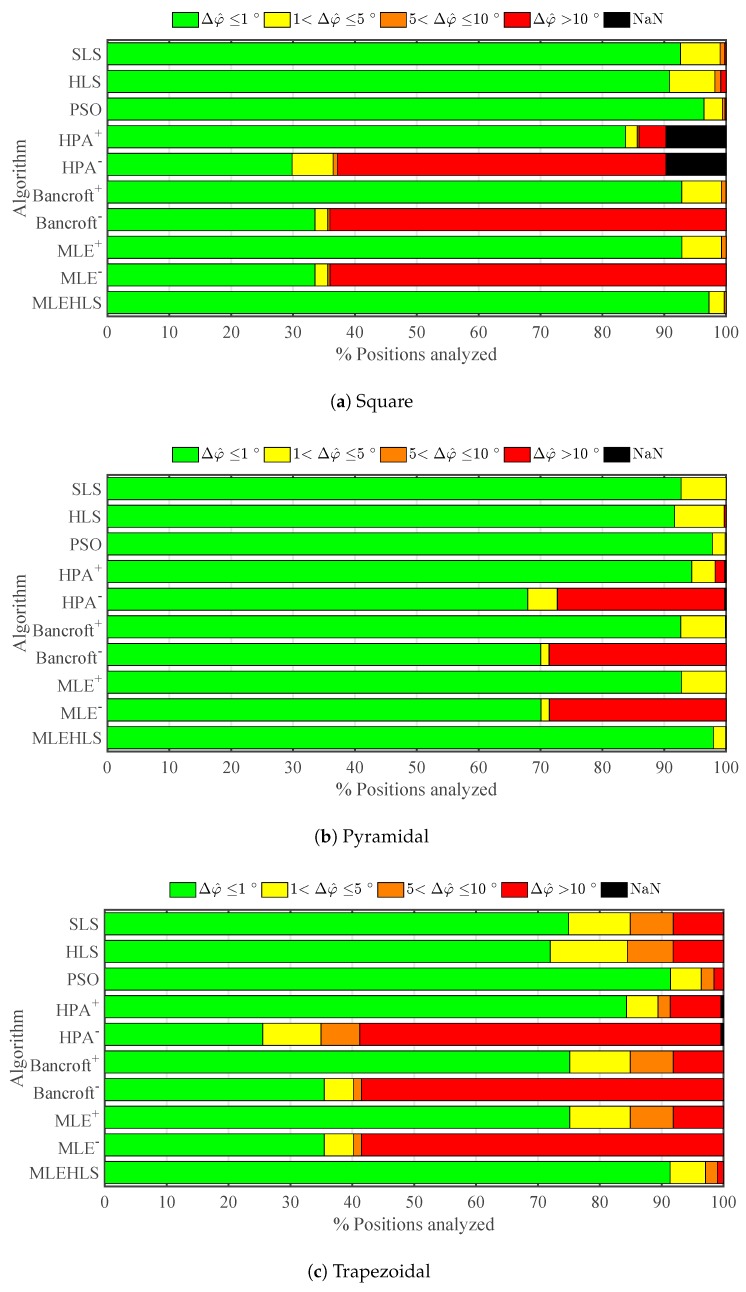
Percentage of positions located within the errors in the azimuth angle $\Delta \hat{\varphi }$ in degrees defined in the legend under digitizing error in time variables for each algorithm and antenna array configuration.

Results for radial distance, elevation angle and azimuth angle under time sampled conditions for the same arrangements as the theoretical simulations are displayed in Figure 8, Figure 9 and Figure 10 and Table 6, Table 7, Table 8 and Table 9. As expected, the accuracy decreases compared with the theoretical case. It may be seen that the radial error is the most critical variable; see Figure 8 and Table 6. Interestingly, the best algorithm appears to be again MLE-HLS with PSO a close second. Both methods localize 49–50% of the analysed points with an average error of less than 20 cm. The pyramidal configuration appears to provide the poorest accuracy, i.e., 37%, whilst the square and trapezoidal configurations provide improved accuracy results, localizing 61–65% of the analysed points with an error lower than 20 cm. In many cases, an average error less than 20 cm may be considered acceptable considering that the maximum distances of the antennas from the source is around 10 m; see Figure 3.

Elevation angle is calculated with good accuracy as seen in Figure 9, with the best two algorithms again being MLE-HLS and PSO. The elevation angle is calculated with lower than or equal to $1^{\circ }$ accuracy for 93% of the analysed positions as shown in Table 7. Further, 97% of the azimuth angle points analysed are calculated with an error lower than or equal to $1^{\circ }$; see Figure 10 and Table 8.

These results indicate that good bearing angle determination is achieved with MLE-HLS and PSO for any of the antenna configurations even under time sampled conditions. However, determining radial distance to the source accurately depends on the algorithm used and also the specific antenna layout employed.

#### 4.2.2. Computational Time

The computational times spent on average by each algorithm for one position calculation are displayed in Table 9. The times are very similar to those evaluated under theoretical conditions for almost all the algorithms. In this case, only the HLS algorithm is slower because the time sampled digitization provokes situations where the algorithm does not converge or has more difficulties in converging.

## 5. Conclusions

An investigation into the performance of source localization algorithms has been presented for the case where four receivers have been considered. It has been shown that the iterative algorithms (SLS, HLS and PSO) require a definition of the initial parameters, which can significantly affect solution accuracy and computational time. Furthermore, PSO includes random variables that can further induce more uncertainty in the repeatability of the results. These algorithms have a considerable disadvantage compared to the non-iterative algorithms (HPA, Bancroft, MLE and MLE-HLS), which provide direct source location solutions using only the receiver antenna positions and the time variables as inputs.

Analysing the results presented in this paper, it can be concluded that every algorithm yields different results in terms of source localization accuracy and computational time. The location accuracy for each algorithm, when time sampled digitization is considered, is as expected poorer than under theoretical high resolution time conditions. The average computational time for each algorithm considered separately is similar for both theoretical and time sampled digitizing situations, except HLS, which runs slower in time sampled conditions due to increased iterative activity in finding the solutions.

The accuracy for each algorithm and various antenna layouts has been investigated as a function of spherical source location coordinates, i.e., radial direction, elevation angle and azimuth angle. Analysing the results, radial distance appears to be the most critical variable having larger errors under time sampled digitization. On the other hand, angular direction is successfully calculated by almost all algorithms for most of the analysed source points. It may also be noted that when the pyramidal antenna arrangement is analysed, the radial estimation has larger errors when compared with the square and trapezoidal arrangements. Conversely, elevation and azimuth angle calculations have smaller errors for the pyramid arrangement.

It has also been demonstrated that the proposed MLE-HLS algorithm yields the best results. This algorithm uses the OF based on HLS, Equation (17), also applied in PSO, to evaluate the correct source solutions. In PSO, the search is conducted in 3D space where there are infinite possible solutions. The main idea of the combined algorithm is to evaluate two unique points from the MLE algorithm, instead of evaluating all possible solutions in 3D space. This limits the search to a more efficient and more effective solution as shown in the presented results.

MLE-HLS locations are highly accurate under theoretical conditions, but in time sampled digitized conditions, the accuracy decreases. When introducing a digitizing error in the time variables, the MLE-HLS accuracy is approximately the same as PSO. Analysing the radial position, MLE-HLS locates 49.9% of the analysed points with an error of less than 20 cm, whilst for PSO, it is 49.2%. Analysing elevation angle, MLE-HLS evaluates 93.8% of positions with an error less than $1^{\circ }$, whilst for PSO, it is 93.6%. In the case of azimuth angle, the percentages are 97.6% for MLE-HLS and 97.1% for PSO. From the results, the accuracy of angle determination from both algorithms with time sampled digitizing is almost the same. Indeed, the direction is therefore estimated effectively in most of the positions studied, i.e., around 93%. On the other hand, the distance estimation is poor; estimated in 50% of the analysed points.

The computational time for the iterative algorithms (SLS, HLS and PSO) is substantially longer than the non-iterative algorithms (HPA, Bancroft, MLE and MLE-HLS). Comparing the computational time on average of the most accurate algorithms, PSO and MLE-HLS, it can be seen that PSO with $3.3\times 10^{-2}$ s on average is slower than MLE-HLS with $1.6\times 10^{-4}$ s. From the work presented in this paper, it is shown that MLE-HLS is slightly superior to PSO in relation to source localization accuracy. Both algorithms provide good accuracy of localization results. Further, MLE-HLS has a much reduced computational time when compared to PSO.

## Figures and Tables

**Table 1:** Antenna positions (m) for the three configurations in the study.

Layout		Ant 1	Ant 2	Ant 3	Ant 4
Square	x	−1.00	−1.00	1.00	1.00
	y	−1.00	1.00	1.00	−1.00
	z	−1.00	1.00	−1.00	−1.00
Pyramid	x	$-\frac{\sqrt{3}}{3}$	$-\frac{\sqrt{3}}{3}$	$2\frac{\sqrt{3}}{3}$	0.00
	y	−1.00	1.00	0.00	0.00
	z	$-\sqrt{\frac{2}{3}}$	$-\sqrt{\frac{2}{3}}$	$-\sqrt{\frac{2}{3}}$	$\sqrt{\frac{2}{3}}$
Trapezoidal	x	−0.66	−0.66	0.66	0.66
	y	−1.00	1.00	2.00	−2.00
	z	−1.00	1.00	−1.00	−1.00

**Table 2:** Percentage of positions analysed located with a radius error $\Delta \hat{r}$ lower than 1 cm under theoretical conditions for each algorithm and antenna array configuration. SLS, standard least squares; HPA, hyperbolic positioning algorithms.

$\Delta \hat{r}\leq 1\;$cm	Square	Pyram	Trapez	Mean
SLS	74.3%	50.2%	67.2%	**63.9%**
HLS	82.3%	81.4%	73.6%	**79.1%**
PSO	90.7%	91.2%	90.4%	**90.8%**
HPA${}^{+}$	79.0%	84.2%	81.5%	**81.6%**
HPA${}^{-}$	12.8%	15.7%	18.2%	**15.5%**
Bancroft${}^{+}$	83.9%	81.4%	73.7%	**79.7%**
Bancroft${}^{-}$	18.6%	18.7%	26.4%	**21.2%**
MLE${}^{+}$	83.9%	81.4%	73.7%	**79.7%**
MLE${}^{-}$	18.6%	18.7%	26.4%	**21.2%**
MLE-HLS	100.0%	100.0%	100.0%	**100.0%**

**Table 3:** Percentage of positions analysed located with an elevation angle error $\Delta \hat{\theta }$ less than $1^{\circ }$ under theoretical conditions for each algorithm and antenna array configuration.

$\Delta \hat{\theta }\leq 1^{\circ }$	Square	Pyram	Trapez	Mean
SLS	84.9%	86.3%	77.5%	**82.9%**
HLS	84.5%	86.6%	77.7%	**82.9%**
PSO	92.7%	95.4%	92.6%	**93.6%**
HPA${}^{+}$	80.8%	89.0%	84.5%	**84.8%**
HPA${}^{-}$	15.6%	21.8%	22.8%	**20.1%**
Bancroft${}^{+}$	85.7%	86.8%	77.7%	**83.4%**
Bancroft${}^{-}$	21.7%	24.3%	30.1%	**25.4%**
MLE${}^{+}$	85.7%	86.8%	77.7%	**83.4%**
MLE${}^{-}$	21.7%	24.3%	30.1%	**25.4%**
MLE HLS	100.0%	100.0%	100.0%	**100.0%**

**Table 4:** Percentage of positions analysed located with an azimuth angle error $\Delta \hat{\varphi }$ lower than $1^{\circ }$ under theoretical conditions for each algorithm and antenna array configuration.

$\Delta \hat{\varphi }\leq 1^{\circ }$	Square	Pyram	Trapez	Mean
SLS	92.8%	92.8%	78.3%	**87.9%**
HLS	91.8%	92.6%	78.6%	**87.6%**
PSO	96.3%	97.9%	92.8%	**95.7%**
HPA${}^{+}$	83.8%	94.4%	84.9%	**87.7%**
HPA${}^{-}$	30.0%	68.2%	24.3%	**40.8%**
Bancroft${}^{+}$	93.0%	92.7%	78.7%	**88.2%**
Bancroft${}^{-}$	33.7%	70.1%	31.1%	**45.0%**
MLE${}^{+}$	93.0%	92.8%	78.7%	**88.2%**
MLE${}^{-}$	33.7%	70.2%	31.1%	**45.0%**
MLE HLS	100.0%	100.0%	99.9%	**100.0%**

**Table 5:** Summary of the mean computational time spent, in seconds, for each position analysed in theoretical conditions by each algorithm.

Algorithm	Square	Pyram	Trapez	Mean
SLS	$7.8\times 10^{-1}$	$1.3\times 10^{0}$	$5.9\times 10^{-1}$	$8.9\times 10^{-1}$
HLS	$2.4\times 10^{-1}$	$2.5\times 10^{-1}$	$9.0\times 10^{-1}$	$1.9\times 10^{-1}$
PSO	$3.3\times 10^{-2}$	$3.4\times 10^{-2}$	$3.2\times 10^{-2}$	$3.3\times 10^{-2}$
HPA	$1.6\times 10^{-5}$	$1.2\times 10^{-5}$	$1.2\times 10^{-5}$	$1.3\times 10^{-5}$
Bancroft	$2.0\times 10^{-4}$	$1.8\times 10^{-4}$	$1.8\times 10^{-4}$	$1.9\times 10^{-4}$
MLE	$9.1\times 10^{-5}$	$8.1\times 10^{-5}$	$8.1\times 10^{-5}$	$8.4\times 10^{-5}$
MLE-HLS	$1.9\times 10^{-4}$	$1.5\times 10^{-4}$	$1.5\times 10^{-4}$	$1.6\times 10^{-4}$

**Table 6:** Percentage of positions located with a radius error $\Delta \hat{r}$ lower than 20 cm under time sampled digitizing error for each algorithm and antenna array configuration.

$\Delta \hat{r}\leq 20\;$cm	Square	Pyram	Trapez	Mean
SLS	57.7%	33.7%	52.9%	**45.7%**
HLS	56.5%	33.4%	52.1%	**45.0%**
PSO	61.4%	37.1%	64.2%	**49.2%**
HPA${}^{+}$	53.6%	33.8%	58.5%	**43.7%**
HPA${}^{-}$	6.5%	6.5%	11.8%	**6.5%**
Bancroft${}^{+}$	58.0%	33.7%	52.6%	**45.8%**
Bancroft${}^{-}$	8.9%	6.8%	18.1%	**7.9%**
MLE${}^{+}$	58.0%	33.7%	52.6%	**45.8%**
MLE${}^{-}$	8.9%	6.8%	18.1%	**7.9%**
MLE HLS	62.2%	37.6%	64.8%	**49.9%**

**Table 7:** Percentage of positions analysed located with an elevation angle error $\Delta \hat{\theta }$ lower than $1^{\circ }$ under digitizing error in time variables for each algorithm and antenna array configuration.

$\Delta \hat{\theta }\leq 1^{\circ }$	Square	Pyram	Trapez	Mean
SLS	84.2%	86.0%	73.9%	**85.1%**
HLS	81.5%	84.7%	71.7%	**83.1%**
PSO	92.2%	94.9%	91.1%	**93.6%**
HPA${}^{+}$	79.8%	88.3%	83.8%	**84.0%**
HPA${}^{-}$	14.5%	21.1%	23.6%	**17.8%**
Bancroft${}^{+}$	84.3%	86.2%	74.0%	**85.3%**
Bancroft${}^{-}$	20.9%	23.5%	33.9%	**22.2%**
MLE${}^{+}$	84.3%	86.2%	74.0%	**85.3%**
MLE${}^{-}$	20.9%	23.5%	33.9%	**22.2%**
MLE HLS	92.8%	94.9%	90.8%	**93.8%**

**Table 8:** Percentage of positions analysed located with an azimuth angle error $\Delta \hat{\varphi }$ lower than $1^{\circ }$ under time sampled digitizing error for each algorithm and antenna array configuration.

$\Delta \hat{\varphi }\leq 1^{\circ }$	Square	Pyram	Trapez	Mean
SLS	92.6%	92.7%	75.0%	**92.7%**
HLS	90.8%	91.7%	72.0%	**91.2%**
PSO	96.4%	97.8%	91.4%	**97.1%**
HPA${}^{+}$	83.8%	94.4%	84.3%	**89.1%**
HPA${}^{-}$	29.8%	67.9%	25.5%	**48.9%**
Bancroft${}^{+}$	92.8%	92.7%	75.2%	**92.7%**
Bancroft${}^{-}$	33.5%	70.0%	35.5%	**51.8%**
MLE${}^{+}$	92.8%	92.8%	75.2%	**92.8%**
MLE${}^{-}$	33.6%	70.1%	35.5%	**51.8%**
MLE HLS	97.2%	97.9%	91.4%	**97.6%**

**Table 9:** Summary of the mean computational time spent, in seconds, for each position analysed under digitizing error in time variables by each algorithm.

Algorithm	Square	Pyram	Trapez	Mean
SLS	$7.6\times 10^{-1}$	$1.3\times 10^{0}$	$5.6\times 10^{-1}$	$8.7\times 10^{-1}$
HLS	$2.2\times 10^{-1}$	$3.2\times 10^{-1}$	$2.1\times 10^{-1}$	$2.5\times 10^{-1}$
PSO	$3.3\times 10^{-2}$	$3.5\times 10^{-2}$	$3.2\times 10^{-2}$	$3.3\times 10^{-2}$
HPA	$1.3\times 10^{-5}$	$1.3\times 10^{-5}$	$1.2\times 10^{-5}$	$1.3\times 10^{-5}$
Bancroft	$1.9\times 10^{-4}$	$1.9\times 10^{-4}$	$1.8\times 10^{-4}$	$1.8\times 10^{-4}$
MLE	$9.1\times 10^{-5}$	$8.2\times 10^{-5}$	$8.1\times 10^{-5}$	$8.5\times 10^{-5}$
MLE HLS	$1.8\times 10^{-4}$	$1.6\times 10^{-4}$	$1.5\times 10^{-4}$	$1.6\times 10^{-4}$
